# LPS-induced inflammation in the chicken is associated with CCAAT/enhancer binding protein beta-mediated fat mass and obesity associated gene down-regulation in the liver but not hypothalamus

**DOI:** 10.1186/1746-6148-9-257

**Published:** 2013-12-17

**Authors:** Yanhong Zhang, Feng Guo, Yingdong Ni, Ruqian Zhao

**Affiliations:** 1 Key Laboratory of Animal Physiology & Biochemistry, Nanjing Agricultural University, Nanjing 210095, P. R. China

**Keywords:** FTO, LPS, Inflammation, Chicken

## Abstract

**Background:**

The fat mass and obesity associated gene (FTO) is widely investigated in humans regarding its important roles in obesity and type 2 diabetes. Studies in mammals demonstrate that FTO is also associated with inflammation markers. However, the association of FTO with inflammation in chickens remains unclear. In this study, male chickens on day 28 posthatching were injected intraperitoneally with lipopolysaccharide (LPS) or saline to investigate whether the FTO gene is involved in LPS-induced inflammation.

**Results:**

We detected significant down-regulation of FTO mRNA in the liver (*P* < 0.01), but not in the hypothalamus, 2 and 24 h after LPS challenge. Toll-like receptor (TLR) 2 (*P* < 0.01) and TLR4 (*P* < 0.01) followed the same pattern as FTO, being suppressed significantly in liver but not in hypothalamus. IL-1β was dramatically up-regulated (*P* < 0.01) in both liver and hypothalamus 2 h after LPS challenge, while activation of IL-6 was observed in the liver (*P* < 0.01), but not in hypothalamus. The 5′-flanking sequence of the chicken FTO gene contains nine predicted binding sites for CCAAT/enhancer binding protein beta (C/EBP beta) and one for signal transducer and activator of transcription 3 (STAT3). Significant elevation of C/EBP beta was detected in the liver (*P* < 0.01), but not in the hypothalamus, 2 h after LPS challenge. Lipopolysaccharide challenge increased the C/EBP beta binding to FTO promoter in the liver (*P* < 0.01 for fragment 1, *P* < 0.05 for fragment 2), although the protein content of C/EBP beta was not altered. Moreover, injection of LPS resulted in enhanced phosphorylation of liver STAT3, a downstream transcription factor in IL-6 signaling. Although phosphorylated STAT3 was not detected to directly bind to FTO promoter, it was found to interact with C/EBP beta.

**Conclusion:**

Our results reveal that FTO expression in liver, but not in hypothalamus, is affected by the i.p. injection of LPS, which may be mediated through tissue-specific FTO transcriptional regulation by C/EBP beta and STAT3 interaction.

## Background

Fat mass and obesity associated gene (FTO) is strongly associated with body mass index and obesity in human genome wide association studies [1]. The role of FTO in obesity has been further confirmed by transgenic manipulation in mice. FTO-deficient mice show reduced fat mass in white adipose tissue [2], while ubiquitous over-expression of FTO leads to increased food intake and fat mass deposition [3]. Obesity is known to be related to chronic subclinical inflammation which plays an important role in the development of type 2 diabetes and cardiovascular diseases. FTO has been implicated to be associated with inflammation [4]. Polymorphism of FTO gene contributes to the variation in plasma level of C-reactive protein, a marker of obesity-associated inflammation [5]. Genetically modified mice with decreased FTO activity exhibit improved inflammatory profile in abdominal white adipose tissue [6].

FTO is ubiquitously expressed in multiple tissues, with high abundance in liver and brain, especially hypothalamus. The liver and hypothalamus are both indispensable in the regulation of energy balance [7]. Hepatic and hypothalamic FTO expression can be affected by feeding status. In mice, FTO mRNA expression was significantly reduced in hypothalamic arcuate nucleus [7], yet increased in the liver [8], in response to fasting. In rats, food deprivation [9] and high fat diet [10] remarkably increase hypothalamic FTO mRNA expression. High fat diet has been found to induce hypothalamic [11] and hepatic [12] inflammation. To date, the association between FTO and obesity has been widely studied, whereas how FTO expression in the liver and hypothalamus is related to inflammation remains elusive.

Lipopolysaccharide (LPS) administration has been used as a good model for studying systemic inflammation [13]. LPS-induced inflammatory response is mediated through Toll-like receptor (TLR) 4, resulting in the expression of proinflammatory cytokines, such as IL-1β and IL-6. FTO was found to be expressed in leukocytes and was drastically upregulated in mouse macrophages in response to the stimulation of interferon gamma and LPS [14]. Nevertheless, the mechanism underlying the inflammatory stimulants-induced FTO expression is still unknown. Numerous transcriptional factors are involved in the process of inflammation, among which are signal transducer and activator of transcription 3 (STAT3) and CCAAT/enhancer binding protein beta (C/EBPβ) [15]. STAT3 signaling pathway is reported to mediate the hypothalamic FTO downregulation during energy restriction in rats [16], whereas FTO may act as a coactivator of C/EBPβ, a master transcriptional regulator of adipogenesis [17].

The expression and function of FTO in chickens received much less attention compared to that in mammals. It has been shown that the profile of FTO expression in chickens is similar to that in mammals [18]. FTO expression was decreased in ventral medial hypothalamus [18], while increased in the liver [19], in response to fasting in the chicken. Broiler chickens reared under commercial condition are threatened by the large amounts of LPS from the dust, and the inhalation of LPS causes the chronic or acute inflammation. In order to elucidate the possible involvement of FTO in the inflammation process, here we use LPS injection as a pro-inflammatory stimulus to examine the alterations of FTO expression in chicken liver and hypothalamus. As the 5′ sequence of chicken FTO gene contains predicted binding sites for both C/EBPβ and phosphorylated STAT3 (p-STAT3), we further investigated the role of these two transcription factors in the regulation of FTO gene expression in response of LPS challenge in the chicken.

## Methods

### Animals and experimental design

Fertile eggs laid by the Yellow Feathered Chicken were obtained from Southern Poultry Breeding Company of WENS Co. Ltd., Guangdong, China. Eggs were incubated in an electric forced-draft incubator at 37.5 ± 0.5°C and 60% relative humidity. On day 28 posthatching, male chickens were selected and randomly divided into control (saline) and LPS groups. Chickens in LPS group were injected intraperitoneally with LPS from Escherichia coli 055:B5 (L2880, Sigma-Aldrich) at a dose of 0.5 mg/kg body weight (BW). Chickens in the control group were injected with the same volume of saline. Two and 24 h after injection, liver and hypothalamus were obtained and frozen in liquid nitrogen and stored at −70°C. The hypothalamus was taken according to the generally accepted standards. Briefly, the hypothalamus was dissected from the ventral surface of the brain. Two transverse cuts were made at the apex of the optic chiasm and the rostral margin of the mammillary bodies. Bilateral cuts were then made 2 mm either side of the midline and the whole hypothalamus was removed according to the chicken brain atlas [20].

All experimental procedures were approved by the Animal Ethics Committee of Nanjing Agricultural University.

### Quantitation of mRNA by real-time PCR

Frozen liver and hypothalamus samples were ground in liquid nitrogen before total RNA extraction with TRIzol reagent (15596026, Invitrogen). Total RNA extracts were then treated with DNase I (D2215, Takara) to eliminate possible contamination of genomic DNA. Two micrograms of total RNA were reverse transcribed and 2 μL of diluted cDNA (1:20) were used for real-time PCR analysis. All the primers were listed in Table 1, and chicken β-actin was selected as a reference gene. The method of 2^-ΔΔCt^ was used to analyze the real-time PCR data. The abundance of mRNA was presented as the fold change relative to the average level of the control group 2 h after LPS challenge.

**Table 1:** Primer sequences used in real-time PCR analysis and ChIP assay

**Target gene**	**Sequence (F: forward, R: reverse)**	**GenBank access**	**Applications**
IL-1β	F: 5′-AACATCGCCACCTACAAG-3′	Y15006	mRNA quantification
	R: 5′-TACTCGGTACATACGAGATGGAAA-3′		
IL-6	F: 5′- CATGGACTGGAGCACAAGTA-3′	AJ309541.1	mRNA quantification
	R: 5′-TGGAGAGCAGCCCATGTAA-3′		
FTO	F: 5′-TGAAGGTAGCGTGGGACATAGA-3′	NM_001185147	mRNA quantification
	R: 5′-GGTGAAAAGCCAGCCAGAAC-3′		
TLR2	F:5′-ATCCTGCTGGAGCCCATTCAGAG-3′	NM_ 204278.1	mRNA quantification
	R:5′-TTGCTCTTCATCAGGAGGCCACTC -3′		
TLR4	F: 5′-GTTTGACATTGCTCGGTCCT-3′	NM_001030693	mRNA quantification
	R: 5′-GCTGCCTCCAGAAGATATGC-3′		
C/EBPβ	F: 5′- 5′-ACGAGGCGGACTGTTTGG-3′	NM_205253	mRNA quantification
	R: 5′- GCTGCTGGGATGCTGCTAA-3′		
STAT3	F: 5′- CAACAACCCCAAGAAC-3′	NM_001030931.1	mRNA quantification
	R: 5′- GCTGAGACCACGCTTT-3′		
β-Actin	F: 5′-TGCGTGACATCAAGGAGAAG-3′	NM_205518	mRNA quantification
	R: 5′-TGCCAGGGTACATTGTGGTA-3′		
Fragment 1	F: 5′- AGTTTGCCTCATGCTGGTA-3′	ENSGALG00000003591	ChIP
	R: 5′- CTGTAGCGAAGTTGGGTGG-3′		
Fragment 2	F: 5′- CCAGGAGTTCCAGCATTA-3′	ENSGALG00000003591	ChIP
	R: 5′- ATGGCTCCATCCAGTTGC-3′		

### Western blot analysis

Protein extracts from frozen liver and hypothalamus samples were prepared as previously described [21]. Protein concentrations were determined with a Pierce BCA Protein Assay kit (23225, Thermo). Thirty micrograms of protein extracts were subjected to electrophoresis on a 10% SDS-PAGE gel, and the separated proteins were transferred onto nitrocellulose membranes (BioTrace, Pall Co., USA). Immunoblotting was performed according to the instructions of the manufacturer for each primary antibody. Anti-C/EBPβ antibody (sc-150, 1:500) was purchased from Santa Cruz Biotechnology; anti-pSTAT3 (9145, 1:2000) and anti-STAT3 antibody (4904, 1:2000) were purchased from Cell Signaling Technology; anti-GAPDH antibody (AP0066, 1:10,000) was purchased from Bioworlde. Finally, the signals were detected by enhanced chemiluminescence (ECL) using the LumiGlo substrate (Super Signal West Pico Trial Kit, Pierce, USA). ECL signals were recorded by an imaging system (Bio-Rad, USA) and analyzed with Quantity One software (Bio-Rad, USA). The content of detected proteins was presented as the fold change relative to the average content of the control group 2 h after LPS challenge.

### Prediction of transcription factor binding sites and Chromatin immunoprecipitation (ChIP) assay

The potential transcription factor binding sites were predicted on the 5′-flanking sequence of the chicken FTO gene, about 3000 bp upstream of the translation start site, by using TRANSFAC database. Nine potential binding sites for C/EBPβ and one for STAT3 were predicted and the binding of these two factors on chicken FTO promoter was verified with ChIP analysis.

ChIP analysis was performed according to our previous publication [22]. Briefly, 200 mg frozen liver samples were ground in liquid nitrogen and washed with PBS containing protease inhibitor cocktail (No. 11697498001, Roche). After crosslinking with 1% formaldehyde, samples were lysed, and chromatin was harvested and sonicated to achieve 300–500 bp fragments. The crude chromatin preparations were pre-cleared with 40 μL protein A/G agarose beads (sc-2003, Santa Cruz Biotechnology), and then incubated with 4 μg of anti-C/EBPβ antibody overnight at 4°C. A negative control was included with normal rabbit IgG (sc-2763, Santa Cruz Biotechnology). Immuno-complexes were captured with the beads and DNA fragments were released by reverse cross-linking at 65°C for 8 h. Purified ChIP DNA was used to amplify the FTO gene promoter sequences by real-time PCR with specific primers (Table 1). ChIP results were calculated relative to the input and presented as the fold change relative to the average value of the control group at 2 h.

### Co-Immunoprecipitation

Two-hundred μg of protein extracts from frozen liver were pre-cleared with 40 μL of protein A/G agarose beads at 4°C for an hour, and then immunoprecipitated with 4 μg of antibodies to C/EBPβ overnight at 4°C. A negative control was included with normal rabbit IgG. The protein complexes were then captured by adding 40 μL of protein A/G agarose beads. Immunoprecipitates were collected and denatured with electrophoresis sample buffer. The samples were finally subjected to the Western bolt analysis.

### Statistical analysis

All statistical analyses were performed with SPSS 17.0 for Windows. All data were expressed as mean ± SEM. For body weight, liver weight, relative quantitative data of gene/protein expression, one-way ANOVA was used to access the effects. For ChIP assay results, a *t* test for independent samples was applied. The level of significance was set at *P* < 0.05 in all the analyses.

## Results

### LPS did not affect body weight, but increased relative liver weight

Symptoms of somnolence, anorexia, adipsia, and ruffling of the feathers were observed in chickens subjected to LPS injection. However, no incidence of death was recorded during the experiment period. LPS injection did not affect the body weight of chickens either 2 h or 24 h after the LPS injection. However, the liver weight relative to body weight increased significantly 2 h (*P* < 0.05) and 24 h (*P* < 0.01) after LPS injection (Table 2).

**Table 2:** Body weight, liver weight and liver index 2 h and 24 h after LPS challenge

**Parameters**	**2 h**		**24 h**	
	**Saline**	**LPS**	**Saline**	**LPS**
Body weight (g)	277.98 ± 4.04	273.91 ± 5.13	288.72 ± 7.43	283.70 ± 8.91
Liver weight (g)	8.61 ± 0.19	9.09 ± 0.24	8.91 ± 0.25	10.97 ± 0.65**
Liver index (%)	3.10 ± 0.07	3.31 ± 0.05*	3.09 ± 0.06	3.86 ± 0.17**

Values are mean ± SEM. *P < 0.05 vs. Saline 2 h; **P < 0.01 vs. Saline 2 h.

### LPS down-regulated mRNA expression of FTO, TLR-4 and TLR-2 in the liver but not hypothalamus

**Figure 1:**
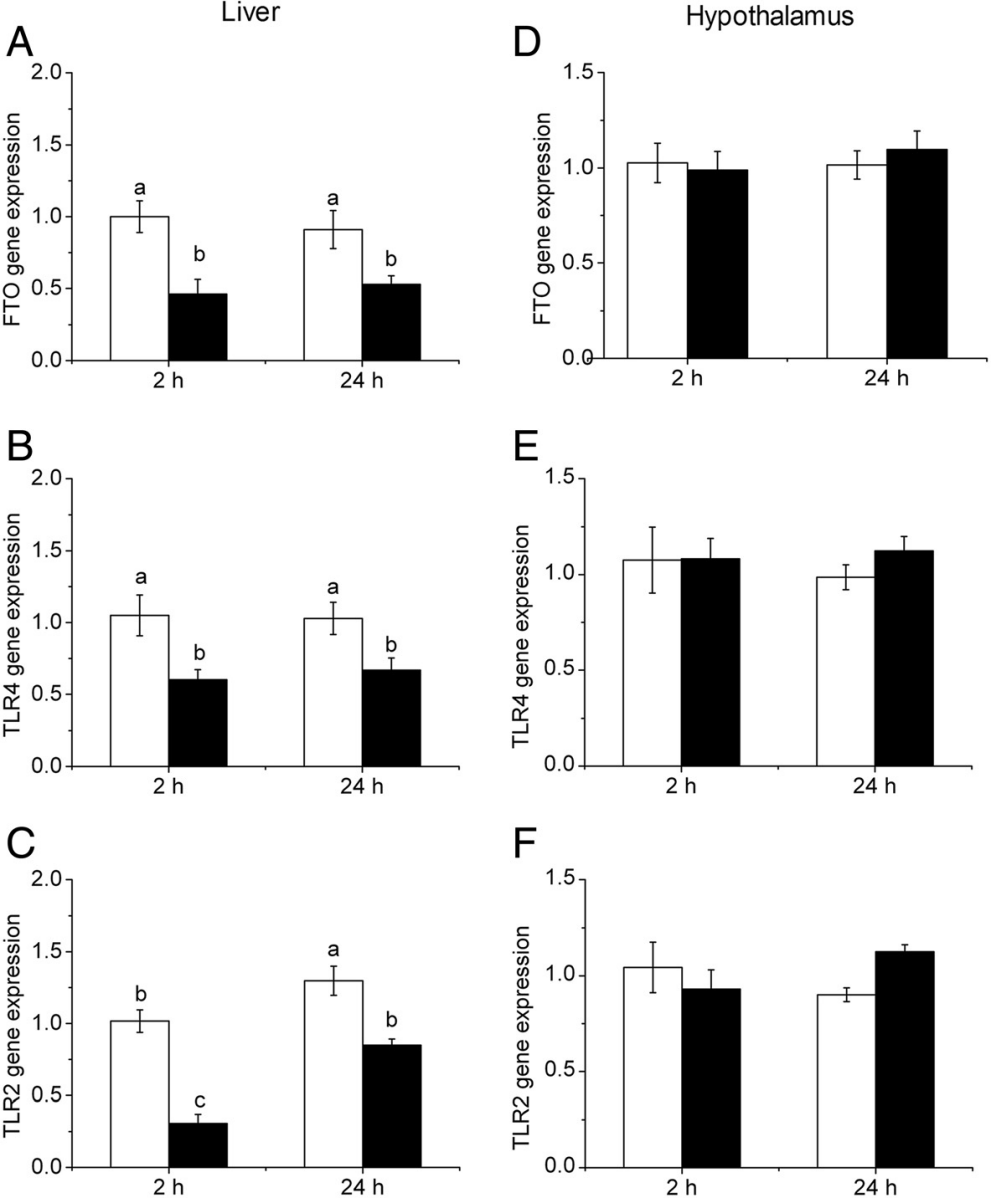
**mRNA expression of FTO (A, D), TLR4 (B, E) and TLR2 (C, F) in liver and hypothalamus after LPS injection.** Values are mean ± SEM, n = 6/group, **□** means control group and **■** is LPS group. Values without common letters are different between two groups, *P* < 0.05.

A tissue-specific response to LPS challenge was observed for FTO, TLR-4 and TLR-2 mRNA levels (Figure 1). Hepatic expression of FTO (Figure 1A), TLR-4 (Figure 1B) and TLR-2 (Figure 1C) was significantly decreased (*P* < 0.05) 2 h and 24 h after LPS injection. In contrast, hypothalamic expression of FTO (Figure 1D), TLR-4 (Figure 1E) and TLR-2 (Figure 1F) was not affected by LPS injection.

### LPS induced expression of both IL-1β and IL-6 in liver, but only IL-1β in hypothalamus

**Figure 2:**
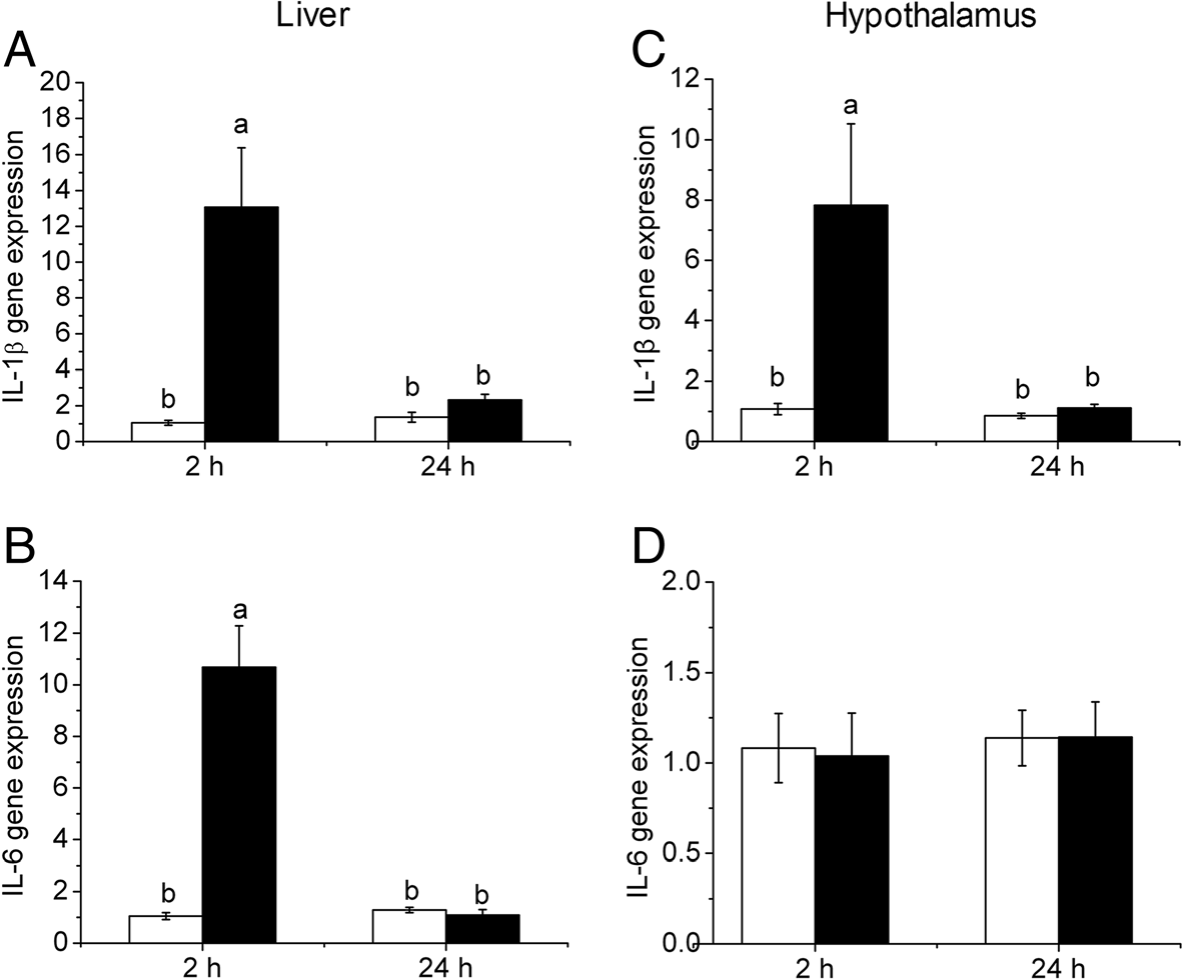
**mRNA expression of IL-1β (A, C) and IL6 (B, D) in liver and hypothalamus after LPS injection.** Values are mean ± SEM, n = 6/group, **□** means control group and **■** is LPS group. Values without common letters are different between two groups, *P* < 0.05.

The mRNA expression of the pro-inflammatory cytokines, IL-1β and IL-6, was determined in liver and hypothalamus to assess the immune responses in respective tissue following LPS challenge (Figure 2). In the liver, both IL-1β (Figure 2A) and IL-6 (Figure 2B) were significantly up-regulated (*P* < 0.01) 2 h after LPS injection, and restored to the basal level 24 h. In hypothalamus, however, only IL-1β (Figure 2C), but not IL-6 (Figure 2D), was activated (*P* < 0.01) 2 h and returned to the control level 24 h after LPS injection.

### LPS up-regulated C/EBPβ mRNA expression in the liver but not hypothalamus

**Figure 3:**
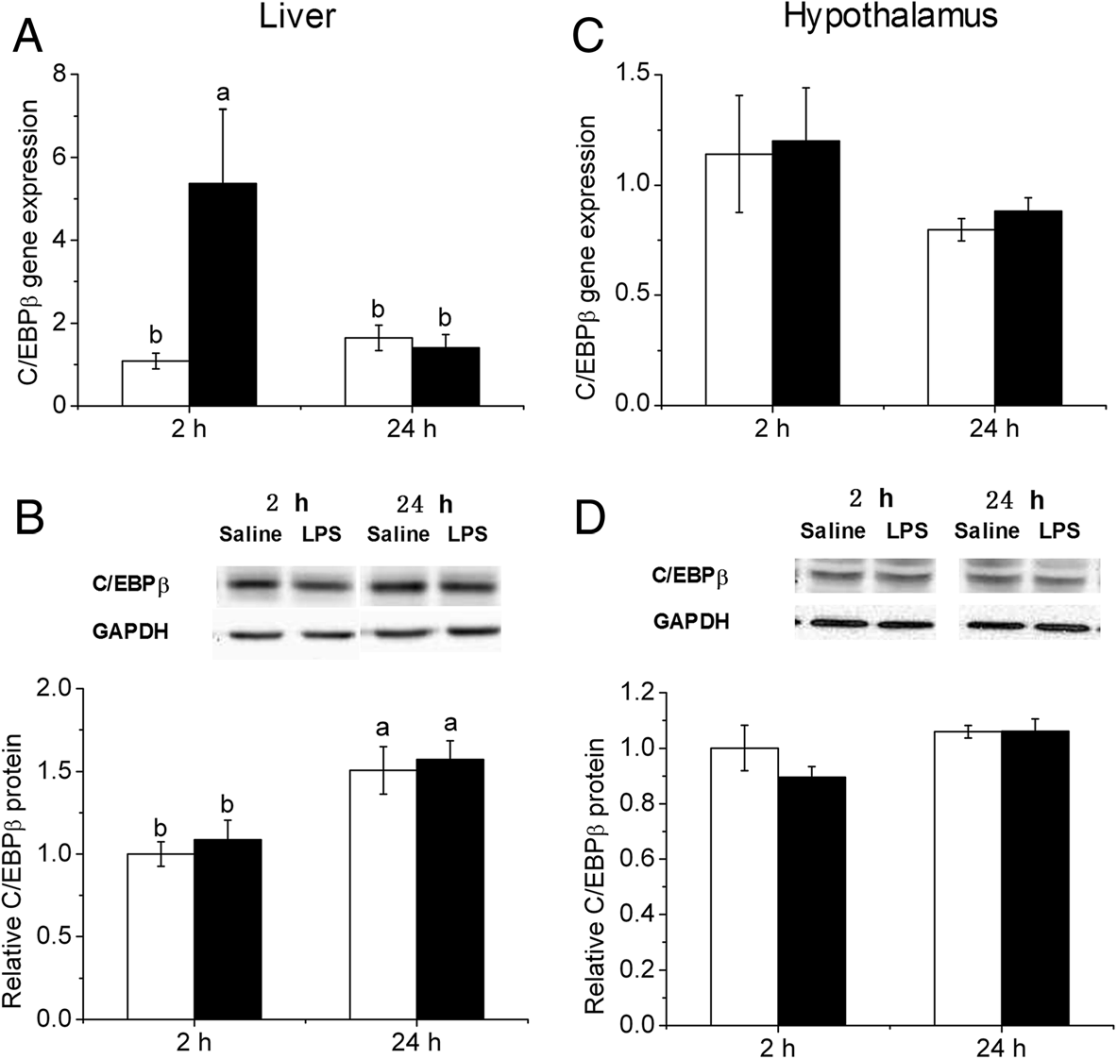
**The expression of mRNA (A, C) and protein (B, D) of C/EBPβ in liver and hypothalamus after LPS treatment.** Values are mean ± SEM, n = 6/group, **□** means control group and **■** is LPS group. Values without common letters are different between two groups, *P* < 0.05.

Hepatic expression of C/EBPβ mRNA was increased significantly 2 h (*P* < 0.01) after LPS injection, and returned to the control level at 24 h (Figure 3A). However, the protein content of C/EBPβ in the liver was not affected by LPS (Figure 3B). In contrast, hypothalamic expression of C/EBPβ mRNA (Figure 3C) and protein (Figure 3D) was not affected by LPS injection.

### LPS induced STAT3 expression and activation in the liver but not hypothalamus

**Figure 4:**
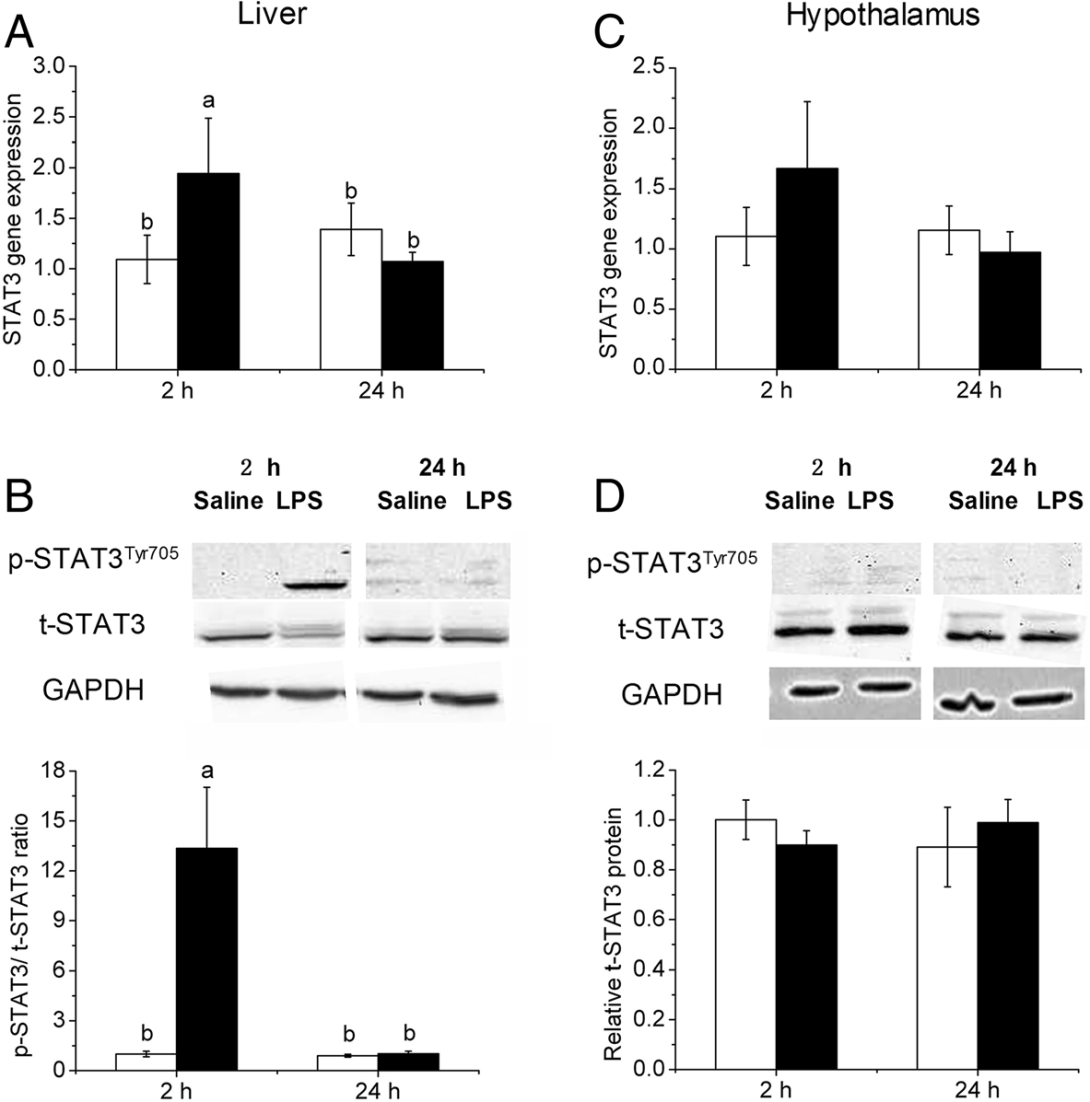
**mRNA expression of STAT3 in liver and hypothalamus (A, C), p-STAT3/t-STAT3 ratio in liver and hypothalamus (B, D) after LPS injection.** Values are mean ± SEM, n = 6/group, **□** means control group and **■** is LPS group. Values without common letters are different between two groups, *P* < 0.05.

In the liver, STAT3 mRNA abundance tended to be higher (*P* =0.056) in LPS-treated chickens 2 h after LPS injection (Figure 4A). The total STAT3 protein content was decreased (*P* < 0.01) 2 h after LPS injection but restored to the control level at 24 h. On the contrary, phosphorylated STAT3 was significantly increased (*P* < 0.01) 2 h after LPS injection. As a result, the ratio of phosphorylated STAT3 (p-STAT3) relative to total STAT3 protein was higher in the liver of LPS-treated chickens (Figure 4B). However, the activation of STAT3 was not detected in hypothalamus, as neither mRNA abundance (Figure 4C) nor protein content (Figure 4D) was affected by LPS injection.

### LPS enhanced C/EBPβ binding to the 5′-flanking region of chicken FTO gene

**Figure 5:**
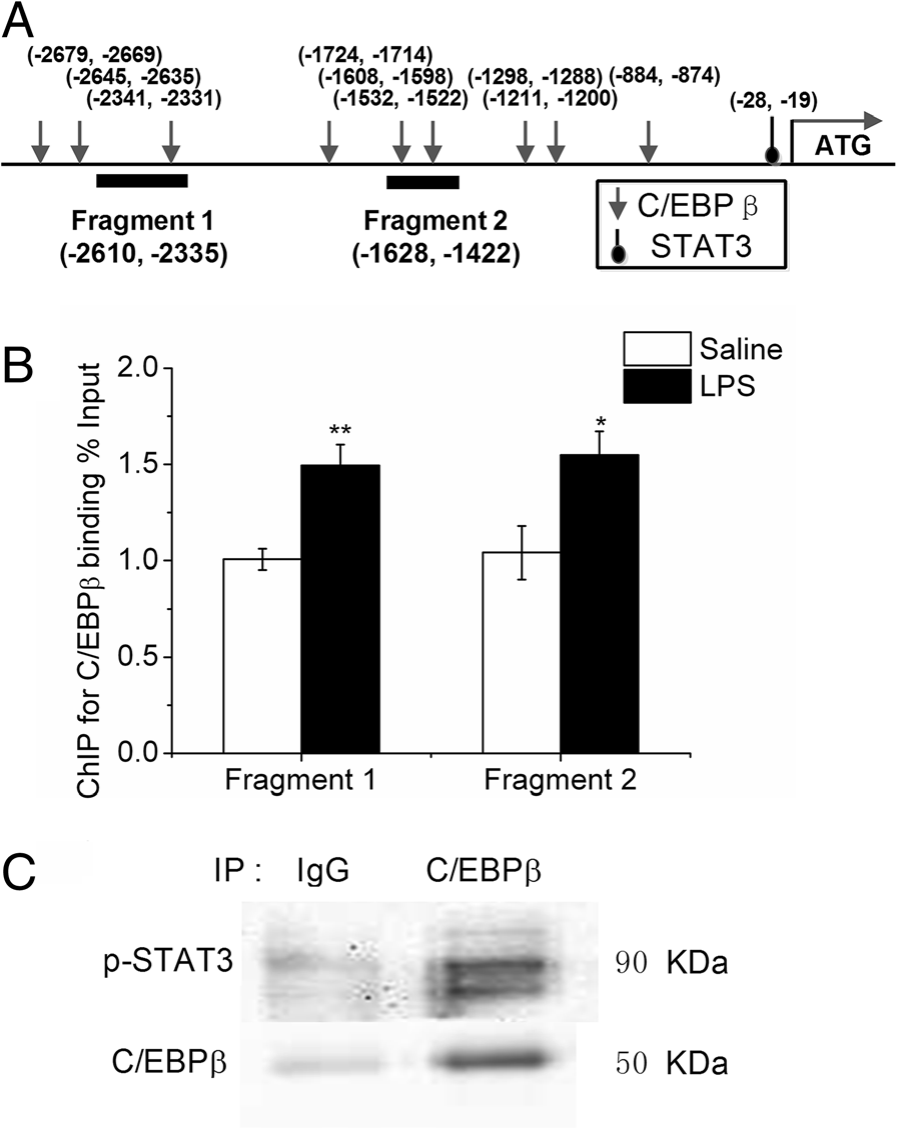
**Schematic structure of chicken FTO gene promoter (A), C/EBPβ binding to FTO gene promoter 2 h after LPS injection (B) and protein-protein interaction of p-STAT3 and C/EBPβ in liver (C).** Values are mean ± SEM, n = 6/group, **□** means control group and **■** is LPS group. *P < 0.05 vs Saline 2 h; **P < 0.01 vs Saline 2 h.

A schematic structure of chicken FTO gene promoter is shown in Figure 5A. Nine C/EBPβ binding sites and one STAT3 binding site were predicted. ChIP assay revealed that C/EBPβ binding to the two FTO gene promoter fragments spanning predicted binding sites was significantly enriched (*P* < 0.01 for fragment 1, *P* < 0.05 for fragment 2) in the liver of LPS-treated chickens at 2 h (Figure 5B). However, no specific binding of STAT3 to chicken FTO promoter was detected, as the binding of p-STAT3 did not differ from that of normal IgG, a negative control (data not shown). Nevertheless, co-immunoprecipitation analysis demonstrated the interaction of p-STAT3 and C/EBPβ in chicken liver.

## Discussion

In this study, we successfully induced systemic inflammation in the chicken by i.p. injection of LPS, which was reflected by the sickness symptoms and remarkably induced expression of inflammatory cytokines in the liver and hypothalamus. IL-1β and IL-6 are regarded as the key pro-inflammation cytokines triggering the acute phase reaction in the liver and modifying the brain-controlled functions such as fever and anorexia. It is reported that LPS-induced expression of pro-inflammatory cytokines such as IL-1β and IL-6 is mediated by TLR4, the recognition receptor of LPS [23]. However, the responses of pro-inflammatory mediators to LPS appear to be different between liver and hypothalamus. Liver is directly exposed to LPS and the Kupffer cells initiate the inflammatory responses by releasing pro-inflammatory cytokines such as IL-1β and IL-6, which communicate with the hepatocytes to induce the acute phase responses [24]. However, hypothalamus is protected by the blood–brain barrier (BBB) from the direct exposure to the cytokines or LPS in the peripheral circulation. Yet, the inflammatory mediators in the circulation can act on the circumventricular organs (CVOs) that lack an effective BBB but contain cells equipped with the TLR4 and receptors for IL-1β and IL-6. The activated immune responses in the CVOs further trigger the inflammation in the other part of brain such as hypothalamus [25]. In this study, we detected the up-regulation of both IL-1β and IL-6 in the liver, but only IL-1β in hypothalamus, in response to LPS injection in the chicken. This divergence in LPS response between liver and hypothalamus may be explained by the above-mentioned differences in signal replay pathways mediating the LPS-induced inflammation in liver and hypothalamus. However, some studies in rodents reported the induction of both IL-1β and IL-6 in hypothalamus following intraperitoneal injection of LPS [26-29]. This discrepancy may attribute to the differences in species, as well as the dose of LPS applied, and/or the timing of the sampling.

Nevertheless, the most interesting finding in this study is the tissue-specific alteration in FTO expression in response to LPS injection. To date, few evidences are available linking FTO with inflammation. For instance, FTO expression in subcutaneous adipose tissue is negatively correlated with the IL-6 gene expression in subcutaneous adipose tissue in morbidly obese German women [30]. However, a cohort study on healthy middle-aged Danish men failed to show the fatness-independent effects of FTO rs9939609 A-allele, a genetic variant most strongly associated with common obesity, on a series of inflammatory markers. Nevertheless, FTO A-allele tended to lower circulating IL-6 level [31]. In the present study, we found negative correlation between FTO and IL-6 expression in chicken liver. LPS-induced hepatic up-regulation of IL-6 gene was found to be associated with a remarkable reduction in FTO expression. Concurrently, FTO expression was not altered in hypothalamus, where IL-6 expression was unaffected. This finding suggests a role of IL-6, but not IL-1β, in the regulation of FTO expression.

Although both IL-1β and IL-6 trigger the acute responses, they activate different protein kinase cascades to accomplish the functions. IL-1β acts predominantly through NF-κB-dependant pathway [32], whereas the role of IL-6 is mainly mediated by Jak/STAT3 pathway [33]. In our study, LPS challenge activated STAT3 signaling in the liver, which was indicated by enhanced STAT3 phosphorylation, despite a decrease in total STAT3. In contrast, similar STAT3 activation was not observed in the hypothalamus, which was in accordance with the lack of IL-6 responses in this brain area. ChIP analysis was employed to detect the direct binding of pSTAT3 to chicken FTO promoter. To our disappointment, however, no direct binding of pSTAT3 to chicken FTO promoter was found. It may not be surprising because only one putative STAT3 binding site was predicted on the 5′-flanking sequence of the chicken FTO gene, about 3000 bp upstream of the translation start site. STAT3 has been found to mediate leptin-stimulated FTO down-regulation in the hypothalamus of rats [16], yet whether STAT3 directly binds to the promoter of FTO remains unexplored. Recently, there are evidences suggesting that STAT3 may participate in the regulation of FTO expression through interacting with other transcriptional factors, such as Cut-like Homeobox 1 (CUX1) [34] which was proven to directly bind to the promoter of FTO gene [35].

Some in vitro studies demonstrated that STAT3 interacts with C/EBPβ [36] to transactivate the promoter of Jun activation-domain binding protein 1 gene [37]. We predicted 9 putative C/EBPβ binding sites on chicken FTO gene promoter; therefore it is reasonable to speculate that STAT3 may regulate FTO expression via protein-protein interaction with C/EBPβ in the process of LPS- induced inflammation. Indeed, hepatic expression of C/EBPβ was significantly up-regulated in response to LPS injection, which is associated with evidently enhanced binding of C/EBPβ to the promoter of chicken FTO gene. Co-immunoprecipitation analysis further confirmed the physical interaction of pSTAT3 and C/EBPβ in chicken liver. However, the contribution of pSTAT3 and C/EBPβ interaction to LPS-induced hepatic FTO repression remains to be verified in the chicken.

Although we provide here the evidence of LPS-induced FTO repression in the liver of chickens, the functional significance for such response is still unknown. Due to the lack of specific antibody against chicken FTO, we were not able to detect changes in FTO protein content. The LPS-induced FTO gene regulation may contribute to the adaptation of energy metabolism in the liver. Additionally, FTO was also reported to be involved in STAT3 or C/EBPβ-mediated inflammatory pathways. Overexpression of FTO remarkably increased STAT3 expression in the arcuate nucleus of the hypothalamus in rats [10] and in the chick embryonic fibroblast cells [38]. Also, FTO was found to act as a transcriptional coactivator to enhance the binding of C/EBPβ to the promoter of target genes [17]. Moreover, FTO has been characterized as a demethylase of N6-methyl-adenosine (m^6^A) which was found widely distributed in the mammalian genes. And genes with this modulation were found to involve in a variety of functional categories including RNA metabolic process and immune system related processes [39]. Whether the response of hepatic FTO to the injection of LPS in chicken was related to the function of being a demethylase is still obscure. The functional relevance of LPS-induced FTO repression in chicken liver as found in the present study remains to be elucidated.

## Conclusion

In summary, the present study demonstrated that FTO gene expression in the chicken liver, but not hypothalamus, was down-regulated by the LPS challenge. IL-6 may act as a mediator regulating the LPS-induced hepatic FTO repression, through the mediation of C/EBPβ and STAT3 interaction. Our findings may help to understand the role of FTO in the LPS-induced inflammatory responses in the chicken.

## Abbreviations

LPS: Lipopolysaccharide; FTO: Fat mass and obesity-associated gene; TLR: Toll-like receptor; STAT3: Signal transducer and activator of transcription 3; C/EBPβ: CCAAT/enhancer binding protein beta.

## Competing interests

The authors declare that they have no financial, personal or professional interests that would have influenced the content of the paper or interfered with their objective assessment of the manuscript.

## Authors’ contributions

YZ performed the experiment and drafted the manuscript. FG helped in ChIP and Co-IP. YN contributed in experiment design and manuscript revision. RZ conceived the idea, designed the experiment, and finalized the manuscript. All authors read and approved the final manuscript.
